# AD Informer Set: Chemical tools to facilitate Alzheimer's disease drug discovery

**DOI:** 10.1002/trc2.12246

**Published:** 2022-04-20

**Authors:** Frances M. Potjewyd, Joel K. Annor‐Gyamfi, Jeffrey Aubé, Shaoyou Chu, Ivie L. Conlon, Kevin J. Frankowski, Shiva K. R. Guduru, Brian P. Hardy, Megan D. Hopkins, Chizuru Kinoshita, Dmitri B. Kireev, Emily R. Mason, Charles T. Moerk, Felix Nwogbo, Kenneth H. Pearce, Timothy I. Richardson, David A. Rogers, Disha M. Soni, Michael Stashko, Xiaodong Wang, Carrow Wells, Timothy M. Willson, Stephen V. Frye, Jessica E. Young, Alison D. Axtman

**Affiliations:** ^1^ UNC Eshelman School of Pharmacy Division of Chemical Biology and Medicinal Chemistry Structural Genomics Consortium Chapel Hill North Carolina USA; ^2^ UNC Eshelman School of Pharmacy Division of Chemical Biology and Medicinal Chemistry Center for Integrative Chemical Biology and Drug Discovery Chapel Hill North Carolina USA; ^3^ Department of Medicine Division of Clinical Pharmacology Indiana University School of Medicine Indianapolis Indiana USA; ^4^ Department of Laboratory Medicine and Pathology University of Washington Seattle Washington USA; ^5^ Institute for Stem Cell and Regenerative Medicine University of Washington Seattle Washington USA

**Keywords:** Alzheimer's disease, drug discovery, target validation, Target Enablement to Accelerate Therapy Development for Alzheimer's Disease

## Abstract

**Introduction:**

The portfolio of novel targets to treat Alzheimer's disease (AD) has been enriched by the Accelerating Medicines Partnership Program for Alzheimer's Disease (AMP AD) program.

**Methods:**

Publicly available resources, such as literature and databases, enabled a data‐driven effort to identify existing small molecule modulators for many protein products expressed by the genes nominated by AMP AD and suitable positive control compounds to be included in the set. Compounds contained within the set were manually selected and annotated with associated published, predicted, and/or experimental data.

**Results:**

We built an annotated set of 171 small molecule modulators targeting 98 unique proteins that have been nominated by AMP AD consortium members as novel targets for the treatment of AD. The majority of compounds included in the set are inhibitors. These small molecules vary in their quality and should be considered chemical tools that can be used in efforts to validate therapeutic hypotheses, but which will require further optimization. A physical copy of the AD Informer Set can be requested on the Target Enablement to Accelerate Therapy Development for Alzheimer's Disease (TREAT‐AD) website.

**Discussion:**

Small molecules that enable target validation are important tools for the translation of novel hypotheses into viable therapeutic strategies for AD.

## 1 INTRODUCTION

Current approved treatments for Alzheimer's disease (AD) address late‐stage symptomatic effects of the disease.^1^, ^2^ The discovery of disease‐modifying interventions that slow or halt progression of AD are critical unmet needs. Promisingly, there has been a recent increase in the number of novel therapeutic strategies targeting AD that have progressed to clinical trials and programs have been developed to foster the discovery of novel targets implicated in AD pathology.^3^

The National Institute on Aging (NIA), recognizing the need to explore new therapeutics for AD, established the Accelerating Medicines Partnership Program for Alzheimer's Disease (AMP AD)^4^ precompetitive partnership among government, industry, and nonprofit organizations in 2014 to transform the existing model for AD diagnostic and drug development. At its inception, the goal of AMP AD was the discovery of novel, clinically relevant therapeutic targets, and development of biomarkers to help validate current therapeutic targets.^5^ AMP AD was initiated as two arms: the Biomarkers in Clinical Trials Project and the Target Discovery and Preclinical Validation Project. The AMP AD Biomarkers Project supported two Phase 2/I secondary prevention trials testing anti‐amyloid therapies with the goal of exploring the utility of tau imaging for tracking responsiveness to treatment and/or disease progression.^5^

The AMP AD Target Discovery and Preclinical Validation Project aimed to accelerate the AD drug discovery process via integration of analyses of large‐scale molecular data from human brain samples with network modeling approaches and experimental validation.^5^ This arm funded six multi‐institutional, multidisciplinary teams who analyzed and integrated multidimensional human “omic” (genomic, epigenomic, RNAseq, and proteomic) data from >2000 human brains at all stages of AD and matched controls with clinical and pathological data.^5^ A fundamental deliverable of this program was open sharing of all data via the AMP AD Knowledge Portal and Agora website.^5^ The types of data generated included novel therapeutic targets for AD, a systems‐level understanding of the networks within which these novel targets operate, and evaluation of their druggability in multiple model organisms.^5^ A round of NIA‐sponsored AMP AD enhancer grants in 2017 augmented the AMP AD data infrastructure and analytical capabilities and enabled generation of new human multi‐omic data from brain, cerebrospinal fluid, and blood samples, as well as single nucleus sequencing data from human and mouse brains.

The NIA‐funded Target Enablement to Accelerate Therapy Development for Alzheimer's Disease (TREAT‐AD) consortium was subsequently started in 2019 to translate AD molecular signatures generated by the AMP AD groups into new treatments.^6^ TREAT‐AD is made up of two multi‐institutional research centers that subscribe to the same open dissemination of all data, research methodologies, and computational and experimental tools.^6^ Building on the >500 nascent candidate AD targets nominated by the AMP AD teams, the TREAT‐AD groups will develop a series of new therapeutic hypotheses centered around a prioritized set of the novel proposed targets and, importantly, deliver a suite of target enabling tools including high‐quality antibodies and chemical probes.^6^ At the inception of TREAT‐AD, two versions of target enabling packages (TEPs) will be created: basic and full.^7^ A basic TEP includes a knockout cell line, validated antibodies, and purified protein related to a target of interest. A full TEP delivers the basic TEP components plus a biochemical and/or biophysical assay, crystal structures, an AD‐relevant knockdown cell line, and initial chemical matter.

High‐quality small molecules are powerful tools that enable interrogation of biological pathways and the exploration of pharmacology in various biological contexts. Through optimization for potent and selective interaction with a specific target in cell‐based assays, the role of a protein in various biological contexts can be studied. The term “chemical probe” has been defined as meeting certain specific criteria, including thresholds related to on‐target potency, family‐wide and sub‐family selectivity, and cell‐based activity.^8^, ^9^ The stringency of requirements to be considered a chemical probe are such that relatively few have been nominated, because each probe demands extensive resources to be designed, optimized, and characterized. Those that have been developed, however, have been profoundly impactful for the research community, especially when released with an open sharing policy.^10^, ^11^, ^12^, ^13^, ^14^, ^15^

The huge investment of resources required to deliver a chemical probe has motivated the generation of chemogenomic and other compound sets. The public kinase inhibitor sets (PKIS/PKIS2) were two early examples of protein‐class targeted sets comprised of published kinase inhibitors that were shared openly alongside all available annotation with the research community.^16^, ^17^ These sets, while not comprised of chemical probes, have been shared with >300 laboratories and have resulted in many new research findings, grants, and publications.^18^ Furthermore, experience with PKIS/PKIS2 informed assembly of the kinase chemogenomic set (KCGS), which includes kinase inhibitors for nearly half of the human kinome that show potent kinase inhibition and a narrow spectrum of activity when screened across a large panel of kinase biochemical assays.^19^ Like PKIS/PKIS2, KCGS is made available with all associated annotation to all interested investigators. Sets of epigenetic modifiers have also been assembled into larger compound libraries that are openly distributed.^9^, ^20^ In this same vein, Boehringer Ingelheim has set up the opnMe program to openly share molecules.^21^ An overarching theme is that the wide‐reaching impact of these small molecules and compound sets is realized due to open sharing, enabling interested investigators to easily request the sets, use them without restrictions, and publish their results.

The Cancer Target Discovery and Development network within the National Cancer Institute (NCI) developed the idea of an “Informer Set.” More than 350 small molecules were assembled and profiled across many human cancer cell lines to reveal dependencies. This Informer Set was comprised of 35 Food and Drug Administration (FDA)‐approved drugs, 54 clinical candidates, and 266 additional tool compounds, which were selected to target distinct nodes in cancer cell circuitry and collectively modulate a broad array of cellular processes. The resultant dataset was deposited into a publicly accessible data portal known as the Cancer Therapeutics Response Portal (CTRP) to be used by other researchers, such as to make connections between the genetic and lineage features of cancer cell lines and small‐molecule sensitivities.^22^ The Informer Set, which is a living resource, has since been expanded to include ≈545 molecules and corresponding data deposited in CTRP. While physical copies of the compound set are not a shared resource, the data within CTRP is immensely useful to the cancer‐focused research community and once again demonstrates the impact of open sharing.^23^

A key deliverable of TREAT‐AD is small molecule tools that enable characterization of the AD targets nominated by AMP AD investigators. While some of these targets are generally underexplored, others are new to AD and thus research results outside of the context of AD have been published. The TREAT‐AD teams will build on and add to the body of knowledge surrounding nascent AD targets nominated by the AMP AD consortium. Facilitated by the chemical tools generated by TREAT‐AD investigators, the AD community can identify those AD‐implicated targets that could be disease modifying and thus transformative.

**RESEARCH IN CONTEXT**

1. **Systematic review**: The authors conducted a comprehensive review of the literature and available databases to identify small molecules that modulate proteins nominated by the Accelerating Medicines Partnership Program for Alzheimer's Disease (AMP AD) as well as other putative targets for Alzheimer's disease (AD) therapy. The primary literature and two databases (Chemical Probes Portal and The Institute of Cancer Research Probe Miner) were queried in assembling the AD Informer Set.
2. **Interpretation**: The study provides a first‐in‐class annotated set of small molecules and associated data for novel targets implicated in AD alongside control compounds that modulate proposed targets in AD.
3. **Future directions**: We created a resource that will accelerate AD‐focused research. The AD Informer Set can be screened by investigators in AD‐relevant assays and/or model systems to illuminate new AD drug discovery opportunities. The availability of associated data for all compounds included will facilitate association of a phenotype with a protein target. The physical AD Informer Set is available through the Target Enablement to Accelerate Therapy Development for Alzheimer's Disease (TREAT‐AD) website.

**HIGHLIGHTS**

- Delivery of an annotated set of 171 small‐molecule modulators targeting 98 proteins.
- Target novel proteins for Alzheimer's disease treatment nominated by Accelerating Medicines Partnership Program for Alzheimer's Disease consortium members.
- Chemical tools that can be used in efforts to validate therapeutic hypotheses.
- Can associate phenotype with protein target using associated compound data.
- A physical copy of the AD Informer Set can be requested via the Target Enablement to Accelerate Therapy Development for Alzheimer's Disease website.

## 2 METHODOLOGY

**FIGURE 1:**
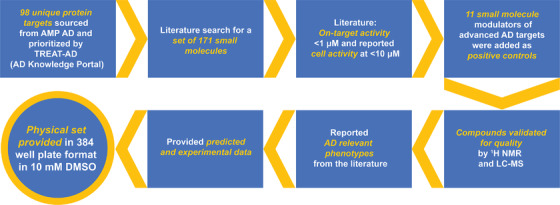
Workflow for assembling the AD Informer Set. Included are compound‐ and gene‐specific details as well as types of data collected for compounds selected for the set. ^1^H‐NMR, proton nuclear magnetic resonance; AD, Alzheimer's disease; AMP AD, Accelerating Medicines Partnership Program for Alzheimer's Disease; DMSO, dimethyl sulfoxide; LC–MS, liquid chromatography–mass spectrometry; TREAT‐AD, Target Enablement to Accelerate Therapy Development for Alzheimer's Disease

To assemble an AD Informer Set of small molecules, protein targets for AD were collected based on AMP AD nominations and those prioritized by the TREAT‐AD teams. Information related to these proteins can also be found within the AD Knowledge Portal and Agora website.^5^ From the start of this collaborative effort between the TREAT‐AD teams, priorities for data collection were agreed upon, duplication was avoided, and data gathering expertise was shared. Next, an exploratory cascade to find small molecules was established, which involved searching the literature and available databases including the Chemical Probes Portal and The Institute of Cancer Research Probe Miner.^9^, ^24^ While there are exceptions, criteria for selection of most small molecules included on‐target biochemical potency, preferably with activity at < 1 μM, and reported cell activity at < 10 μM. It is important to note that the compounds selected for inclusion do not all meet the quality criteria for chemical probes because this would have been overly restrictive given the less‐explored nature of many nominated targets. These small molecules have been characterized as inhibitors, substrates, activators, agonists, or antagonists and should be treated as modulators of a biological target implicated in AD. Like the NCI Informer Set, these compounds are in various stages of development, ranging from only in vitro studies; to use in animals; to approved drugs. To supplement these more investigational small molecules, an additional set of small molecule modulators of advanced AD targets was added as positive controls. These clinical phase compounds were included as more extensively characterized examples that have been used to validate therapeutic hypotheses and for which phenotypic data has already been published. Reported AD‐relevant phenotypes for the targets of these positive control compounds as well as for all other protein targets where they could be found are incorporated into the annotation for the AD Informer Set. Predicted and experimentally collected data for the entire set is also included. Finally, all AD Informer Set compounds have been validated for quality by proton nuclear magnetic resonance and liquid chromatography–mass spectrometry and registered with this data within the University of North Carolina Laboratory Information Management System within the Center for Integrative Chemical Biology and Drug Discovery. Figure 1 captures our workflow in assembling the set and details about the compounds selected.

## 3 RESULTS

The AD Informer Set is a physical set of high‐quality compounds with associated data that can be used by the scientific community to interrogate AD‐implicated biology. The set comprises 171 small molecules that target 98 unique proteins. Where possible, more than one compound preferably built upon differing chemotypes was selected as a modulator of each protein target. Table 1 captures all 98 genes with associated molecules included in the AD Informer Set. Unsurprisingly, proteins that have been more widely studied yielded more available chemical tools, whereas those within the lesser studied proteome provided fewer choices. This resulted in one to seven compounds being included for each target, with at least two for more than half (51) of the 98 protein targets in the set. A total of 11 positive control compounds that are currently in Phase 2/3 clinical trials or already approved drugs were selected for the AD Informer Set (noted in supporting information annotation file and Table 1). Including these positive control compounds results in a distribution of chemical tools in terms of stage of development. Within the AD Informer Set, 36 compounds are approved drugs, 40 are in clinical trials (Phases 1–4), 68 have advanced into animal‐based studies, and 27 have not been explored beyond cellular studies. For all 171 compounds in the set, we have performed and included in accompanying annotation files prediction of blood–brain barrier (BBB) permeability and likelihood of efflux, experimentally determined kinetic solubility measurements, and single‐concentration (10 μM) results in cell‐based microglial viability and phagocytosis assays.

**TABLE 1:** Nomination details for 98 AD‐implicated genes with an associated compound in the AD Informer Set

Gene	# of compounds in AD Informer Set targeting protein product of gene	Nominating group in AMP AD or TREAT‐AD	Nomination input considered by AMP AD team
ACE	2	Duke	Genetics, metabolome
ACHE	2^a^	TREAT‐AD	N/A
ALK	6	Mayo‐UFL‐ISB	RNA
APP	1^a^	TREAT‐AD	N/A
AXL	1	TREAT‐AD	N/A
BACE1	1^a^	TREAT‐AD	N/A
BACE2	1^a^	TREAT‐AD	N/A
BCHE	1^a^	TREAT‐AD	N/A
BCL2	3	Chang Lab	Genetics, RNA
CAPN2	4	Emory	Protein
CDK18	2	Chang Lab	Genetics, RNA
CDK9	1	TREAT‐AD	N/A
CETP	1	Duke	Genetics, metabolome
CHRM2	1^a^	TREAT‐AD	N/A
CRHR1	2	Mayo‐UFL‐ISB	RNA
CTSH	1	TREAT‐AD	N/A
CYP19A1	2	Duke	Genetics, metabolome
CYP3A43	1	Duke	Genetics, metabolome
DHTKD1	1	Duke	Genetics, metabolome
DOCK1	1	Chang Lab	Genetics, RNA
ENO1	1	Emory	Protein
EPHA5	1	Duke	Genetics, metabolome
EPHX2	5	TREAT‐AD	N/A
ERBB3 (HER3)	3	Broad‐Rush‐Columbia, Mayo‐UFL‐ISB	Genetics, RNA
ESRRG	4	Duke	Genetics, metabolome
EZR	2	TREAT‐AD	N/A
FADS1	1	Duke	Genetics, metabolome
FADS2	2	Duke	Genetics, metabolome
FKBP5	3	Mayo‐UFL‐ISB	RNA
FLT3	2	TREAT‐AD	N/A
FOXO1	2	Duke	Genetics, metabolome
GJA1	1	MSSM	Genetics, RNA
HSPA2	2	Chang Lab	Genetics, RNA
HSPB1	3	Broad‐Rush‐Columbia, Emory	Clinical, protein
HSPB2	3	Broad‐Rush‐Columbia, Emory	Clinical, protein
IL6ST	2	Chang Lab	Genetics, RNA
INPP5D	2	Duke, Mayo‐UFL‐ISB, MSSM	Genetics, metabolome, RNA
INPPL1	2	Mayo‐UFL‐ISB	RNA
IRS1	1	Duke	Genetics, metabolome
KAT2B	1	Chang Lab	Genetics, RNA
KCNJ3	3	Duke	Genetics, metabolome
KDM3B	1	Chang Lab	Genetics, RNA
KIT	1^a^	TREAT‐AD	N/A
KPNB1	3	Chang Lab	Genetics, protein
Kv1.3	3	TREAT‐AD	N/A
LXRa	2	TREAT‐AD	N/A
LXRb	2	TREAT‐AD	N/A
LYN	1^a^	TREAT‐AD	N/A
MAPK1 (ERK2)	5	Emory	Protein
MAPK11	1^a^	TREAT‐AD	N/A
MAPK14	1^a^	TREAT‐AD	N/A
MAPK3 (ERK1)	5	Emory	Protein
MAPT	1^a^	TREAT‐AD	N/A
MARK4	3	Duke	Genetics, metabolome
MDK	2	Mayo‐UFL‐ISB	RNA
MERTK	1	TREAT‐AD	N/A
MMP17	1	Duke	Genetics, metabolome
NLRP3	2	TREAT‐AD	N/A
NR3C1	3	Mayo‐UFL‐ISB	RNA
NUPR1	1	Broad‐Rush‐Columbia	Genetics, RNA
P2 × 7	1	TREAT‐AD	N/A
p75NTR	1^a^	TREAT‐AD	N/A
PADI2	1	Emory	Protein
PDCD1	2	Mayo‐UFL‐ISB	RNA
PDE3A	1^a^	TREAT‐AD	N/A
PDE3B	1^a^	TREAT‐AD	N/A
PDGFRα	1^a^	TREAT‐AD	N/A
PDGFRβ	1^a^	TREAT‐AD	N/A
PFKP	1	Chang Lab	Genetics, RNA
PHGDH	4	Broad‐Rush‐Columbia	Clinical, protein
PLEC	1	Broad‐Rush‐Columbia, Chang Lab, Emory	Clinical, genetics, protein
PPARD	1	TREAT‐AD	N/A
PPARG	2	TREAT‐AD	N/A
PRDX6	1	Chang Lab, Emory	Genetics, protein, RNA
PREX1	1	Broad‐Rush‐Columbia, Chang Lab	Genetics, RNA
PRKAR2B	2	Emory	Protein
RPS6KA2 (RSK3)	4	Broad‐Rush‐Columbia	Clinical, protein
RXRA	7	Duke	Genetics, metabolome
S100A4	1	Mayo‐UFL‐ISB	RNA
SCN7A	2	Duke	Genetics, metabolome
SCN9A	2	Duke	Genetics, metabolome
SENP1	1	Chang Lab	Genetics, RNA
SFRP1	2	Mayo‐UFL‐ISB	Proteomics
SGPL1	1	Duke	Genetics, metabolome
SIG‐1R	2^a^	TREAT‐AD	N/A
SLC1A2	1	Chang Lab	Genetics, RNA
SLCO1A2	3	Mayo‐UFL‐ISB	RNA
SREBF1	2	Duke	Genetics, metabolome
SREBF2	1	Duke	Genetics, metabolome
SRR	1	Duke	Genetics, metabolome
STARD10	1	Chang Lab	Genetics, RNA
STAT3	2	Mayo‐UFL‐ISB, MSSM	Genetics, RNA
SYK	4	MSSM	Genetics, RNA
TGFBR2	2	Mayo‐UFL‐ISB, MSSM	Genetics, RNA
TNFRSF1A	2	Chang Lab, MSSM	Genetics, RNA
UNC119B	1	Chang Lab	Genetics, RNA
VCP	4	Emory	Protein
YAP1	3	MSSM	Genetics, RNA

Abbreviations: AD, Alzheimer's disease; AMP AD, Accelerating Medicines Partnership Program for Alzheimer's Disease; Broad, Broad Institute; Chang Lab, Chang Lab at University of Arizona; Columbia, Columbia University; Duke, Duke University; Emory, Emory University; ISB, Institute for Systems Biology; Mayo, Mayo Clinic; MSSM, Mount Sinai School of Medicine; N/A, not applicable; Rush, Rush University; TREAT‐AD, Target Enablement to Accelerate Therapy Development for Alzheimer's Disease; UFL, University of Florida.

Notes:

^a^ Positive control compound.

### 3.1 Physical set access and use specifics

The AD Informer Set will be provided to interested investigators as a single 384‐well plate containing 1 μL of each compound as a 10 mM stock in dimethyl sulfoxide (DMSO). A plate map, included as supporting information, will be provided when the plate is shipped. All information about requesting the set can be found on the TREAT‐AD website: https://treatad.org/data‐tools/ad‐informer‐set/. To obtain the AD Informer Set, prospective users must agree to terms of a materials transfer agreement (MTA), the most significant requirement of which is that data produced from its use be made publicly available. The MTA is provided for consideration without modification. Requests for the set are welcomed from all interested investigators.

### 3.2 Data annotation

**FIGURE 2:**
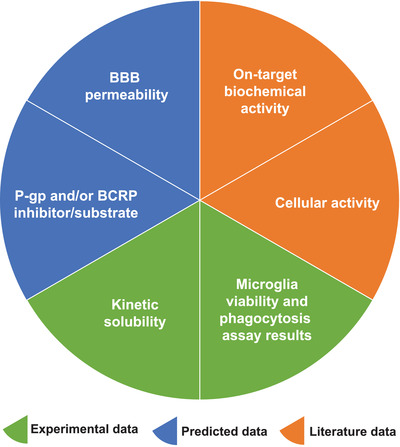
Summary of data annotation collected for the AD Informer Set. Data are subdivided by the source from which it was obtained: experimental, predicted, or literature.

Annotation for the entire set is included as supporting information. This spreadsheet is alphabetized by gene and has been divided into two sections: compound‐specific information and gene‐specific information. A second tab on this spreadsheet defines each column and summarizes the contents provided, including what is meant by certain abbreviations. Within the compound‐specific information section, there is compound identification data including the assigned UNC number, associated lot ID, an alternative/common name, CAS number, vendor that provided the solid sample, SMILES string, and designation as a positive control (where applicable). Also within this subsection is data related to predicted BBB penetration by the StarDrop software program (logBB)^25^, ^26^, ^27^, ^28^ as well as by the ADMET Predictor software program:^29^, ^30^, ^31^ qualitative likelihood of crossing BBB and logarithm of the brain/blood partition coefficient (logBB). Other predicted compound properties included from the ADMET Predictor software program are whether the compound is a likely P‐glycoprotein (P‐gp) and/or breast cancer resistance protein (BCRP) substrate and/or inhibitor, making it susceptible to efflux. Also included is literature‐reported data for the compounds, including on‐target biochemical potency (in nM), whether the compound demonstrates cell activity when dosed at < 10 μM, stage of development and dosing information, kinetic solubility (in μM), and single‐concentration microglial viability and phagocytosis assay activity. PubMed IDs and/or the appropriate database have been included as references for the potency and dosing information assembled. The data annotation provided, and the various sources of this data are summarized in Figure 2.

Within the gene‐specific information portion of this spreadsheet is the Ensembl gene ID(s), published AD‐relevant phenotypes associated with gene and/or its protein product, predicted AD therapeutic direction, and nomination details. Most genes were nominated by AMP AD investigators, while others have been prioritized by TREAT‐AD team members including those selected as positive controls. PubMed IDs have been included as references for AD‐relevant phenotypes referenced within the file. Several of the details related to the genes within this spreadsheet can be cross‐referenced on the Agora website, which can be searched by Ensembl ID or gene symbol. This invaluable resource can be used to find additional specifics surrounding target nomination, evidence of association with AD, expression profiles within the brain, and predicted druggability.

### 3.3 Kinetic solubility determination

Analyses were executed using 10 mM DMSO stocks of AD Informer Set compounds in aqueous buffer at neutral pH (7.4) by Analiza, Inc via total chemiluminescent nitrogen determination as previously described.^32^ Because this method relies on nitrogen detection for quantification, no kinetic solubility value could be determined for compounds lacking nitrogen in their structure (16 in total). All calculated solubility values, after correction, are included in the supporting information annotation file.

### 3.4 Microglial viability and phagocytosis studies

All AD Informer Set compounds were tested in two immortalized microglial cell lines, murine (BV2), and human (HMC3), in a high‐content imaging assay in duplicate by the Chu lab. Experimental details and all data can be found as supporting information files. A few compounds were observed to have inherent fluorescence (noted in the supporting information files).

## 4 DISCUSSION

Nearly all the protein targets nominated by AMP AD represent nascent targets in AD. The intended uses of the AD Informer Set include: (1) to interrogate target validity in established and emerging AD models, (2) to serve as positive controls/comparators versus new chemical entities for AD, and (3) to qualify newly developed or established AD‐relevant assays. Neuroinflammation, for example, is a phenotype that is increased early in AD and, because of its chronic nature, is considered pathogenic.^33^, ^34^ Based on its known role in propagating AD pathology, neuroinflammation is a phenotype that could be investigated using the AD Informer Set. This set is not comprised of highly optimized, potent, and selective small molecule chemical probes,^8^, ^9^ and results generated when using the set should be treated accordingly. In particular, selectivity assessment is limited for many compounds and pharmacological results should be cross‐validated wherever possible by genetic methods of target manipulation.^35^ Most compounds contained herein were optimized within certain biological contexts for applications other than the treatment of AD and many remain unoptimized. Thus, the set should be used to explore related pharmacology within the context of AD. AD Informer Set compounds could then serve as chemical starting points in medicinal chemistry campaigns focused on delivering AD‐specific tools. Optimized tools that are validated to potently engage their target and show a response in an AD‐relevant model would have translational value and/or yield meaningful results in pharmacogenetic studies. The inclusion of 11 positive control compounds in the set, which are in Phase 2/3 clinical trials or FDA‐approved drugs developed for AD, allows benchmarking in specific AD phenotypic assays related to the pathways for which these advanced compounds were developed (see supporting information and Table 1).

As acknowledged above, we have not acquired target class selectivity for the compounds included in the AD Informer Set, and they likely lack the requisite selectivity embodied by chemical probes. As AD is a complex and multifactorial disease and current therapies available show limited ability to modify the disease, the approach of multi‐target drug design is being pursued to simultaneously modulate multiple targets.^36^ Thus, the polypharmacology likely elicited by AD Informer Set compounds in phenotypic assays may reveal phenotypes worthy of further investigation.

The AD Informer Set provides investigators the ability to execute an unbiased screen of compounds that modulate targets implicated in AD. By providing biochemical potency and classification of a compound as cell‐active at 10 μM, we are hoping to enable researchers to use the set effectively. These values are meant to serve as guidelines for selecting a concentration or dose‐response range at which to profile the compounds to ensure on‐target activity. This does not represent all the information that users may want, especially because cell‐based data likely has been acquired in different and/or not relevant cells. Cellular target engagement data, which will be essential for association of a phenotype with a protein target and ideally generated in a relevant system, could not be included for all compounds but is likely contained in provided references for some targets. After an initial screen, we suggest following up on hit compounds to confirm target engagement via monitoring activity of downstream targets and/or through implementing available cellular target engagement assays where they have been developed, such as for kinases.^37^ Via pilot studies that use the AD Informer Set and resultant publications, the body of knowledge surrounding AD Informer Set compounds and targets in the public domain will grow. These will inform other interested users, reduce the likelihood of redundant experiments, and fill in data gaps with relevant findings.

At the beginning of this section, we suggested three ways that AD Informer Set could be used. As an example of interrogating target validity in established and emerging AD models, one could use neurons derived from human induced pluripotent stem cells (iPSC) that harbor an AD‐relevant mutation, such as a line carrying the point mutation in the Presenilin 1 gene known to cause familial AD, and isogenic control iPSC as an AD‐relevant system. Inhibitors of EPHX2 were reported to inhibit tau hyperphosphorylation and neuroinflammation in differentiated SH‐SY5Y human neuroblastoma cells.^38^ A similar study could be executed with the AD Informer Set, which contains five different EPHX2 inhibitors, in the proposed iPSC‐based system to validate EPHX2 as a target in this patient‐derived AD model system. Alternatively, if a high‐quality chemical probe were to be developed for a protein target within the AD Informer Set, then this chemical probe could be screened in the same AD‐relevant assay as the AD Informer Set. This side‐by‐side study would illuminate whether specific modulation of that single protein yields the same result as less selective compounds and thus if polypharmacology is potentially beneficial. In this way, the AD Informer Set would serve as positive controls/comparators versus new chemical entities for AD. Finally, the AD Informer Set could be used to qualify newly developed or established AD‐relevant assays. We describe in the next section screening in a newly established microglia phagocytosis assay that demonstrates this application and confirms utility of the set when considering published results related to specific targets.

As proof‐of‐concept and to demonstrate its utility, we have analyzed the set in cell‐based assays involving immortalized mouse and human cells. Results have motivated the design of follow‐up studies and defined hypotheses about the pathways regulated by these genes as discussed below. Because the set is comprised of modulators that have differential effects on their target(s), including inhibition or activation, it is anticipated that some will transiently phenocopy AD pathology while others may offer a new therapeutic direction to pursue.

**FIGURE 3:**
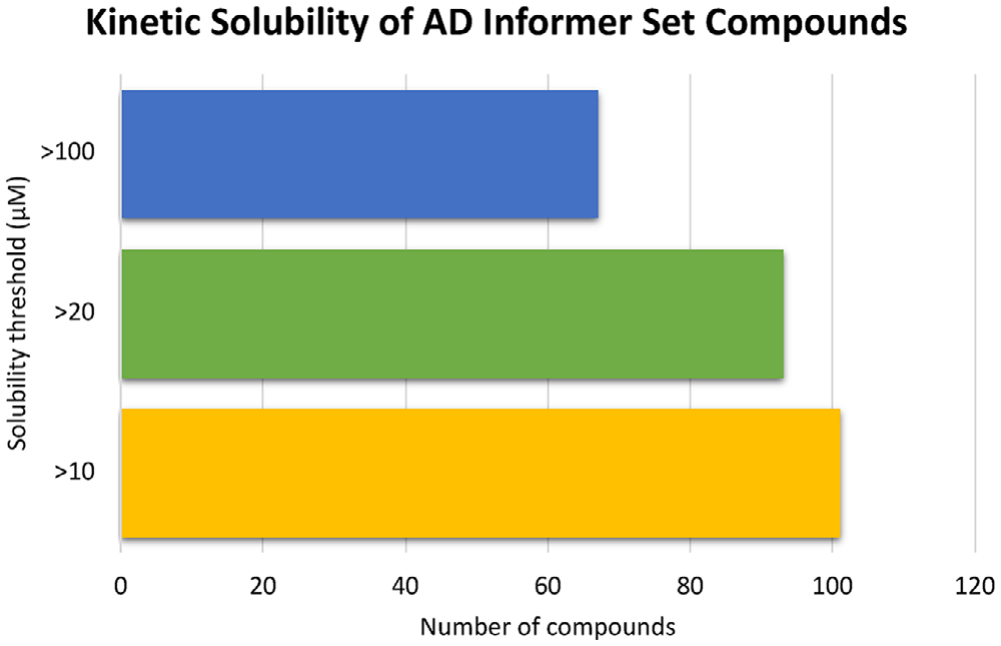
Summary of kinetic solubility data trends for AD Informer Set compounds. Compounds have been binned according to kinetic solubility thresholds to display those that meet minimal (> 10 μM), acceptable (> 20 μM), or desirable (> 100 μM) levels. The most soluble compounds satisfy criteria to be included in all categories. This analysis was done for the 155 compounds in the set that contain nitrogen and thus could be experimentally evaluated.

**FIGURE 4:**
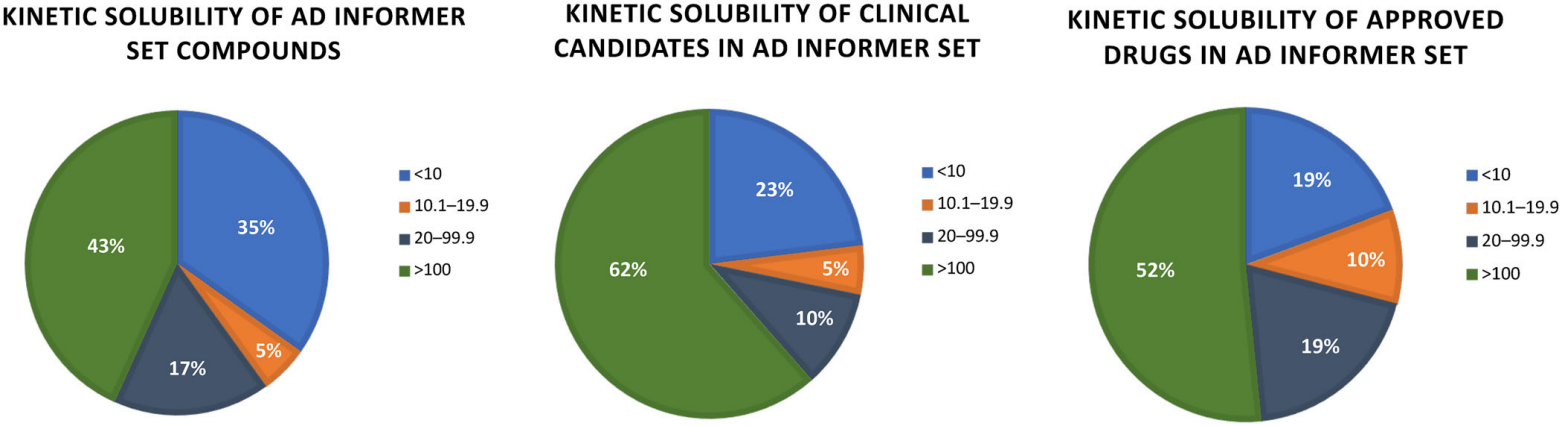
Analysis of kinetic solubility of all compounds as well as subsets of compounds in the AD Informer Set. The 155 compounds in the set for which experimental kinetic solubility was determined were binned into those with poor solubility (< 10 μM), intermediate solubility (10.1 to 19.9 μM), acceptable solubility (20 to 99.9 μM), and desired solubility (> 100 μM). The same thresholds were used to categorize compounds within the AD Informer Set for which kinetic solubility data was collected that are in clinical trials (39 in total) and those that are approved drugs (31 in total).

Beyond simply highlighting those compounds that will likely precipitate in aqueous buffers or media, the kinetic solubility study results, summarized in Figures 3 and 4, provide general insights. As nearly all compounds (101 of 155) demonstrated solubility > 10 μM, most compounds in the AD Informer Set are not considered poorly soluble. For reference, during a medicinal chemistry optimization program a kinetic solubility concentration > 20 μM is often considered acceptable (93 of 155 compounds fit this criteria), while a concentration > 100 μM is desirable (67 of 155 compounds fit this criteria). While drugs with low water solubility are predisposed to low and variable oral bioavailability and there are structural modifications known to improve aqueous solubility, ≈40% of currently marketed compounds remain poorly water soluble.^39^, ^40^, ^41^ As further support that approved drugs do not have to fit a certain kinetic solubility concentration, 6 of 31 approved drugs in the AD Informer Set that were evaluated have kinetic solubility values < 10 μM and 15 of 31 have kinetic solubility values < 100 μM (Figure 4). The same analysis was done for AD Informer Set compounds currently in clinical trials as well as the entire set (Figure 4). It has been suggested that kinetic solubility may also play a minor role in passive diffusion across the BBB.^42^

Microglial viability studies demonstrated that most compounds in the set are not toxic. We chose to profile the AD Informer Set in both human (HMC3) and murine (BV2) immortalized microglia. While most compounds in the set were likely optimized in a human context, available in vivo model systems for AD are non‐human. We expect that there will be target differences between humans and these model systems. Our use of both HMC3 and BV2 cell lines highlights that there are cell line–to–cell line as well as species‐to‐species differences in target expression and pathway connectivity.

The role of microglia in AD has been well documented and was thus chosen as a cell type in which to profile the AD Informer Set.^43^, ^44^, ^45^ As an example, neuroinflammation, as detected by the presence of activated complement proteins, interleukins, cytokines, and chemokines in microglia and astrocytes, is increased early in AD and is considered pathogenic due to its chronic nature.^33^, ^34^ Cytotoxic effects can be assessed by considering multiple parameters. A cell count < 70% control (lower number, more toxic) is the simplest measurement. More nuanced details come from considering nuclear area measurement (< 80% control indicates toxicity) and nuclear DNA intensity, where > 120% control is indicative of early apoptosis and < 70% is indicative of later apoptosis. Considering cell count alone and excluding fluorescent compounds, 33 (20%) of 168 compounds resulted in cytotoxicity at 10 μM in human microglial cells, while 65 (39%) resulted in cytotoxicity at 10 μM in mouse microglial cells. Except for three compounds, all compounds that were toxic to human microglial cells were also toxic to mouse microglial cells. This included several compounds targeting protein kinases (ALK, CDKs, MARK4, and SYK), FOXO1, and VCP. Gratifyingly, of the 11 positive control compounds, only a compound with observed red fluorescence (UNC10303097A/TRx0237 [LMTX] mesylate) that likely interfered with the assay manifested toxicity in both microglial cell lines. UNC10126573A/masitinib displayed cytotoxicity only in mouse microglia. The remainder of compounds optimized for clinical use for AD did not exhibit cytotoxicity, which supports that optimization of the chemical leads in the AD Informer Set can reduce toxicities resulting from off‐target liabilities.

**FIGURE 5:**
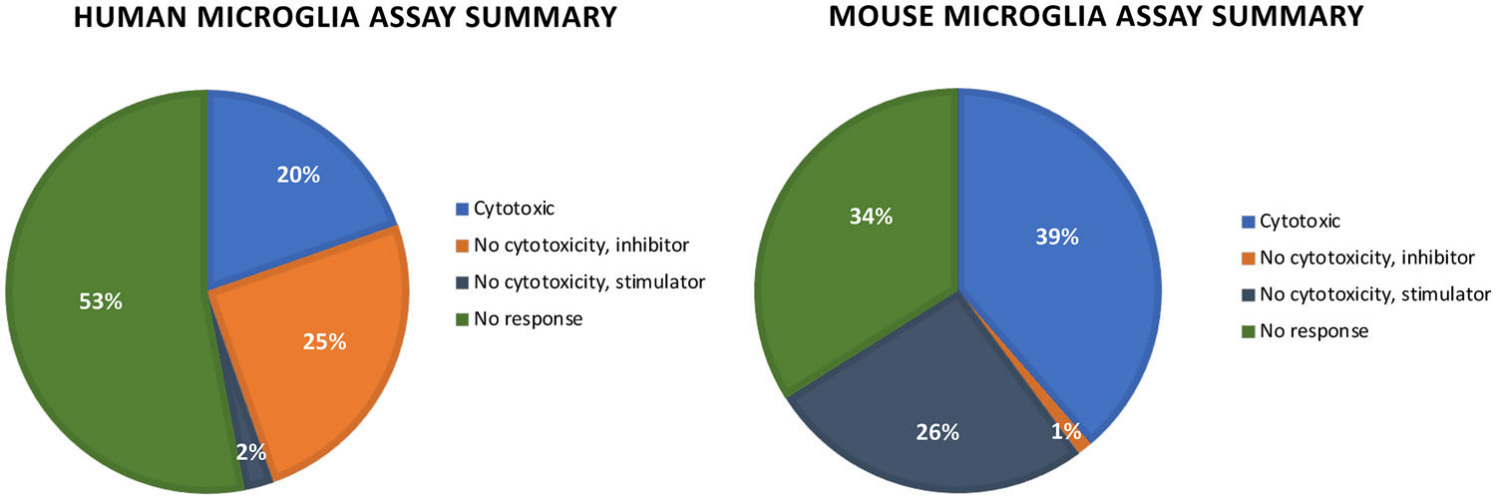
Summary of results from microglia viability and phagocytosis activity assays. Data are presented according to the cell line used: human (HMC3) or murine (BV2). Compounds that were cytotoxic (cell count < 70% control) were categorized separately. For compounds that were not considered cytotoxic, those that elicited inhibition (< 70% control) or stimulation (> 150% control) were captured. Finally, the remaining compounds that were not cytotoxic but also did not induce either inhibition or stimulation were grouped in a no‐response category. Only 168 of the 171 compounds tested were included in this analysis, as the remaining three compounds demonstrated fluorescence that could interfere with the assay results.

For phagocytosis, < 70% control was considered inhibition, while > 150% control was considered stimulation. It is important to note that stressed cells, such as those that are dying, are prone to show an increase in phagocytosis. Considering the phagocytosis results in human microglial cells for non‐fluorescent compounds, many more were inhibitory (69) than stimulatory (7). If we further exclude compounds that demonstrated cytotoxicity (cell count < 70% control), 42 compounds (25%) were inhibitory, while four compounds (2%) acted as stimulators of microglial phagocytosis at 10 μM. Considering the phagocytosis results in mouse microglial cells, the opposite trend was observed: many more non‐fluorescent compounds were stimulatory (68) than inhibitory (33). Considering only non‐cytotoxic compounds that lacked fluorescence at 10 μM, only two (1%) were found to inhibit and 44 (26%) stimulated microglial phagocytosis in murine BV2 cells. These results are summarized in Figure 5. Efforts are under way to understand these differences in the response of human (HMC3) and murine (BV2) cells to treatment to pick the best cell line to use in assays moving forward. Only one compound (UNC10302867A/MRT67307) was a stimulator of phagocytosis in both human and mouse microglia, while many compounds were inhibitors of phagocytosis in both human and mouse microglia. Considering the published literature that connects genes with phagocytosis in the context of AD, DOCK1 protein (UNC8367A/TBOPP) has been reported to play a role in phagocytosis and was found to be stimulatory in human microglia at 10 μM in our assay as well.^46^ MerTK promotes phagocytosis and thus MerTK inhibition is proposed to lead to inhibition of phagocytosis.^47^ In accordance with this hypothesis, MerTK inhibitor UNC2025C (same as UNC2025)^48^ inhibited mouse and human phagocytosis in our assay at 10 μM.

This first iteration of the AD Informer Set is available immediately to the research community. We anticipate supplementing the set and releasing additional compounds with associated data as part of future iterations. New small molecules will be developed from our TREAT‐AD efforts dedicated to delivering chemical probes for nominated AD targets as well as by the larger community. The AD Informer Set is a complement to traditional drug discovery programs and will benefit from these programs in its future iterations. We encourage interested individuals to use the set, publish their results, and we will do our best to aid in data deconvolution, analysis, and further annotation of this resource.

## CONFLICTS OF INTEREST

Jeffrey Aubé has received compensation from the American Chemical Society, Elsevier Beillstein, Pergament & Cepeda, and the National Institutes of Health as well as consulting fees from the University of Kansas and an honorarium from Case Western Reserve University. He has also served as NIH study section chair twice in the past 36 months and held an unpaid leadership role for the ACS Division of Organic Chemistry. Kevin J. Frankowski received support as a speaker from the Gordon Research Conference. Timothy I. Richardson is an advisor for Enveda Biosciences. Xiaodong Wang received an honorarium and travel support from University of Pittsburgh. Stephen V. Frye received consulting fees from Artios, Astex, GSK‐Crick, Cullgen, Design Therapeutics, Flare, Larkspur, Mitokinin, Pathios, ReViral, Meryx, and eFFector. He has also received honoraria from NIEHS, Scripps, St. Jude, Emory/Winship Cancer Center, Oregon Health Sciences University, University of New Mexico Comprehensive Cancer Center, University of Lexington, and University of Utah. Jessica E. Young received travel reimbursements from AAIC, Columbia University, and Duke University. Jeffrey Aubé, Ivie L. Conlon, Kevin J. Frankowski, Dmitri B. Kireev, Timothy I. Richardson, Xiaodong Wang, Carrow Wells, Timothy M. Willson, Stephen V. Frye, and Alison D. Axtman disclose that they have patents planned, issued, or pending within the past 36 months but that there is no overlap of these patents with the work described herein. All funding provided to the institution and individual authors has been disclosed in the funding information and the declaration of interest section. No other authors have conflict of interests to disclose.

## Supporting information

Supporting information

Supporting information

Supporting information

Supporting information
