# Supplementary material for: AD Informer Set: Chemical tools to facilitate Alzheimer's disease drug discovery
Source: Alzheimers Dement (N Y). 2022 Apr 20;8(1):e12246. doi: 10.1002/trc2.12246 (PMC9019904; doi:10.1002/trc2.12246)
Supplement: Supplementary file 4 — Supporting information [file TRC2-8-e12246-s002.pdf]

Experimental details

Microglial viability and phagocytosis studies: The pHrodo-myelin phagocytosis/cell viability assay, modified based on a published method [1], was run with immortalized BV2 (mouse microglia) and HMC3 (human microglia) cell lines in the Chu lab at IUSM. The 384-well plate high content analysis assay with BV2 and HMC3 microglial cell lines is used to quantify both phagocytosis and cell viability simultaneously. High content imaging is performed with the ArrayScan automatic imaging system and imaging analysis done with the software on the system. After allowing cells to adhere overnight, they are treated with compounds for total 48 hours including final 20 hours seeded with pHrodo-myelin (labeled phagocytosis ligand, purified from mouse brain), then cells are stained with Hoechst-33342 one hour before the imaging (approximately 22 hours after pHrodo-myelin addition). Three measurements are extracted from the assay: 1) phagocytosis signal by mean total phagocytosis spot intensity per cell, 2) total cell counts per well as the main measurement of cell viability, and 3) mean average nuclear intensity per cell as profiling of cell health since apoptotic cells showing nuclear intensity increase (early apoptosis) and decrease (later apoptosis), respectively. These measurements allow for evaluation of compound-induced phagocytosis (1) and cytotoxicity (2 and 3). Assays were carried out at a single concentration (10 µM). Idelalisib (10 µM) and cytochalasin-D (4 µM) were used as positive controls in the assay. All data, which was an average of 2 data points, was normalized to untreated wells and reported as % control, which is included in the Supporting Information both as a raw data file and summarized in the compound/gene-annotation file.

[1] Andreone BJ, Przybyla L, Llapashtica C, Rana A, Davis SS, van Lengerich B, et al. Alzheimer's-associated PLCγ2 is a signaling node required for both TREM2 function and the inflammatory response in human microglia. Nat Neurosci. 2020;23:927-38.
